# Tiny, ornamented eggs and eggshell from the Upper Cretaceous of Utah represent a new ootaxon with theropod affinities

**DOI:** 10.1038/s41598-021-89472-1

**Published:** 2021-05-11

**Authors:** Sara E. Oser, Karen Chin, Joseph J. W. Sertich, David J. Varricchio, Seung Choi, Jeffrey Rifkin

**Affiliations:** 1 Museum of Natural History, University of Colorado, Boulder, CO 80309 USA; 2 Department of Geological Sciences, University of Colorado, Boulder, CO 80309 USA; 3 Department of Earth Sciences, Denver Museum of Nature & Science, Denver, CO 80205 USA; 4 Department of Earth Sciences, Montana State University, Bozeman, MT 59717 USA; 5 University of Colorado, Boulder, CO 80309 USA

**Keywords:** Evolution, Zoology

## Abstract

A new Cretaceous ootaxon (eggshell type) from the Kaiparowits Formation of Grand Staircase-Escalante National Monument is among a growing number of very small eggs described from the Mesozoic. Analyses of two partial eggs (~ 17.7 mm in diameter) and 29 eggshell fragments reveal that this new ootaxon exhibits nodose ornamentation with distinctive branching pore canals that open atop the nodes. Its two-layered microstructure consists of a mammillary layer and a continuous layer with rugged grain boundaries between calcite grains. Although the exact identity of the egg producer is unknown, the eggshell microstructure and small size is consistent with a small-bodied avian or non-avian theropod. The specific combination of small egg size, branching pores, two-layered microstructure, and dispersituberculate ornamentation preserved in this new ootaxon is unique among theropod eggs. This underscores that both eggshell and skeletal fossils of Cretaceous theropods can display a mosaic of transitional morphological and behavioural features characteristic of both avian and non-avian taxa. As such, this new ootaxon increases the diversity of Cretaceous eggs and informs our understanding of the evolution of theropod eggshell microstructure and morphology.

## Introduction

Since its designation in 1996, Grand Staircase-Escalante National Monument (GSENM) in southern Utah has protected a wealth of archaeological, biological, geological, and paleontological resources. The Upper Campanian Kaiparowits Formation exposed within GSENM preserves a spectacular record of Late Cretaceous biota, including many taxa undescribed from other, coeval formations. The Kaiparowits Formation additionally preserves an abundant and diverse assemblage of fossil eggshell^1–4^ including two partial eggs representing a new ootaxon among the smallest Mesozoic eggs described. The small size and unique combination of features present in this new ootaxon are of particular interest because the study of fossil eggshell offers numerous insights into the evolution and reproductive biology of extinct amniotes^5^.

**Figure 1:**
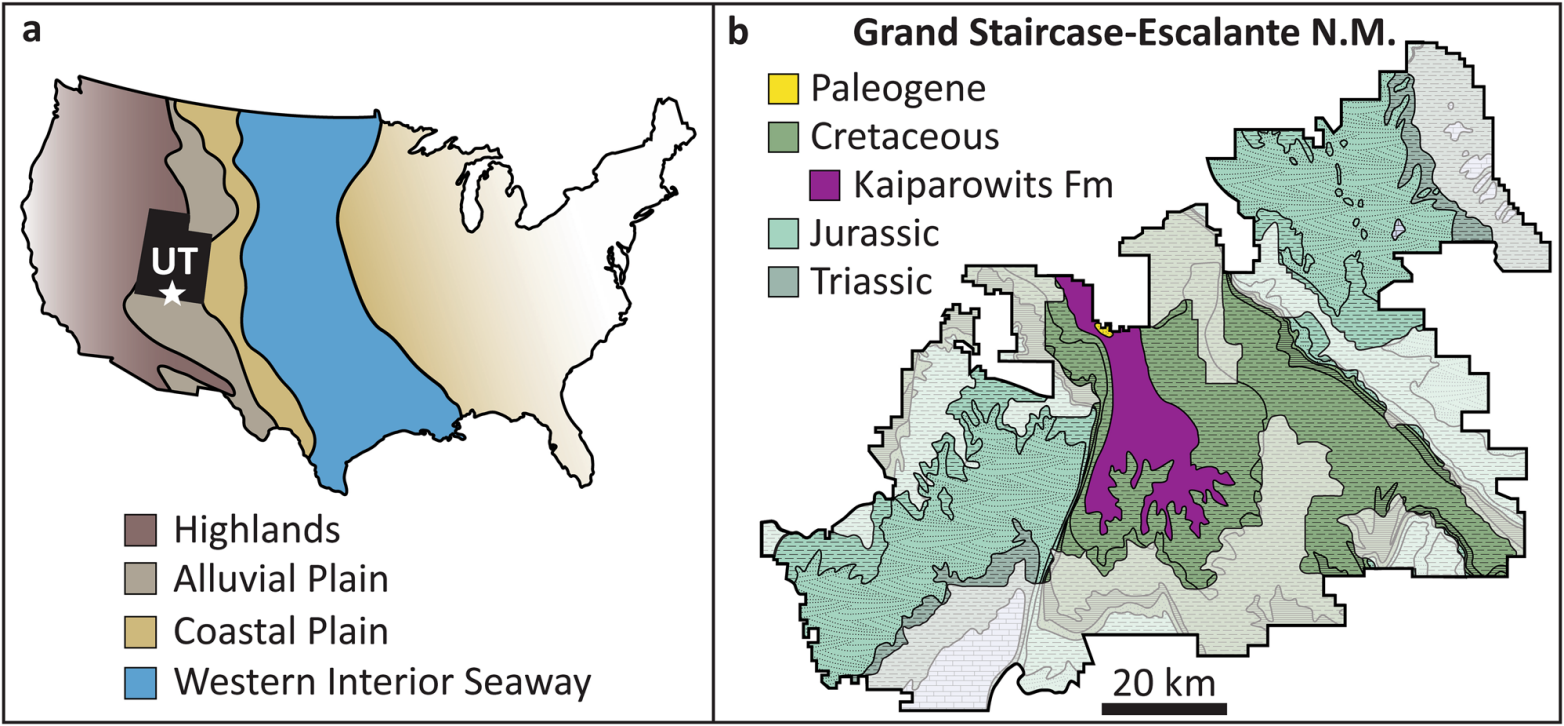
(**a**) Paleogeography of the United States during the Campanian (after Roberts and Kirschbaum, 1995^17^; image generated with Adobe Illustrator CS4, version 14.0.0—see https://www.adobe.com/products/illustrator.html for recent versions). Star denotes location of Grand Staircase-Escalante National Monument. (**b**) Geologic map of GSENM with Cretaceous Kaiparowits Formation highlighted (after Foster et al.^1^; image generated with Adobe Illustrator CS4, version 14.0.0—see https://www.adobe.com/products/illustrator.html for recent versions). Areas shaded white indicate areas of the monument lost to the 2017 boundary modifications of White House Proclamation 6920.

The ~ 860 m-thick succession of sandstone, siltstone, and mudstone of the Kaiparowits Formation exposed within GSENM was deposited on an alluvial plain to the west of the Cretaceous Western Interior Seaway (Fig. 1). Radiometric dates from bentonite deposits place the Kaiparowits Formation in the Campanian between 76.46 and 74.69 Ma^6^. Basin subsidence combined with abundant sediment supply likely supported a high sedimentation rate of approximately 41 cm/ka^7^. A seasonal, subhumid to subtropical paleoenvironment is inferred for the Kaiparowits Formation based upon isotopic studies, leaf margin analysis, and hydromorphic paleosol features (e.g., poor horizonation, slickensides, high organic content, and shallow carbonized root traces) which suggest seasonally waterlogged soils and mild pedogenesis^7–10^. In addition, fossils recovered from floodplain deposits indicate perennial lake and wetland habitats that supported a diverse community of aquatic and semi-aquatic plants, invertebrates, and vertebrates including amphibians, turtles, fish, and crocodyliforms^1,6,8,11–13^. Terrestrial taxa preserved within the Kaiparowits include lizards, snakes, mammals, pterosaurs, ceratopsians (e.g., *Utahceratops, Kosmoceratops, Nasutoceratops*), hadrosaurs (e.g., *Gryposaurus*, *Parasaurolophus*), nodosaurids, ankylosaurids (e.g., *Akainacephalus*), pachycephalosaurids, orodromines, the tyrannosaurid *Teratophoneus curriei*, dromaeosaurids, troodontids (e.g., *Talos sampsoni*), *Ornithomimus*, the oviraptor *Hagryphys giganteus*, and enantiornithine birds including *Mirarce eatoni*^14–16^.

**Systematic palaeontology**

Oofamily Incertae sedis

Oospecies ***Stillatuberoolithus storrsi*** oogen. et oosp. nov.

(Figs. 2, 3, 4)

**Figure 2:**
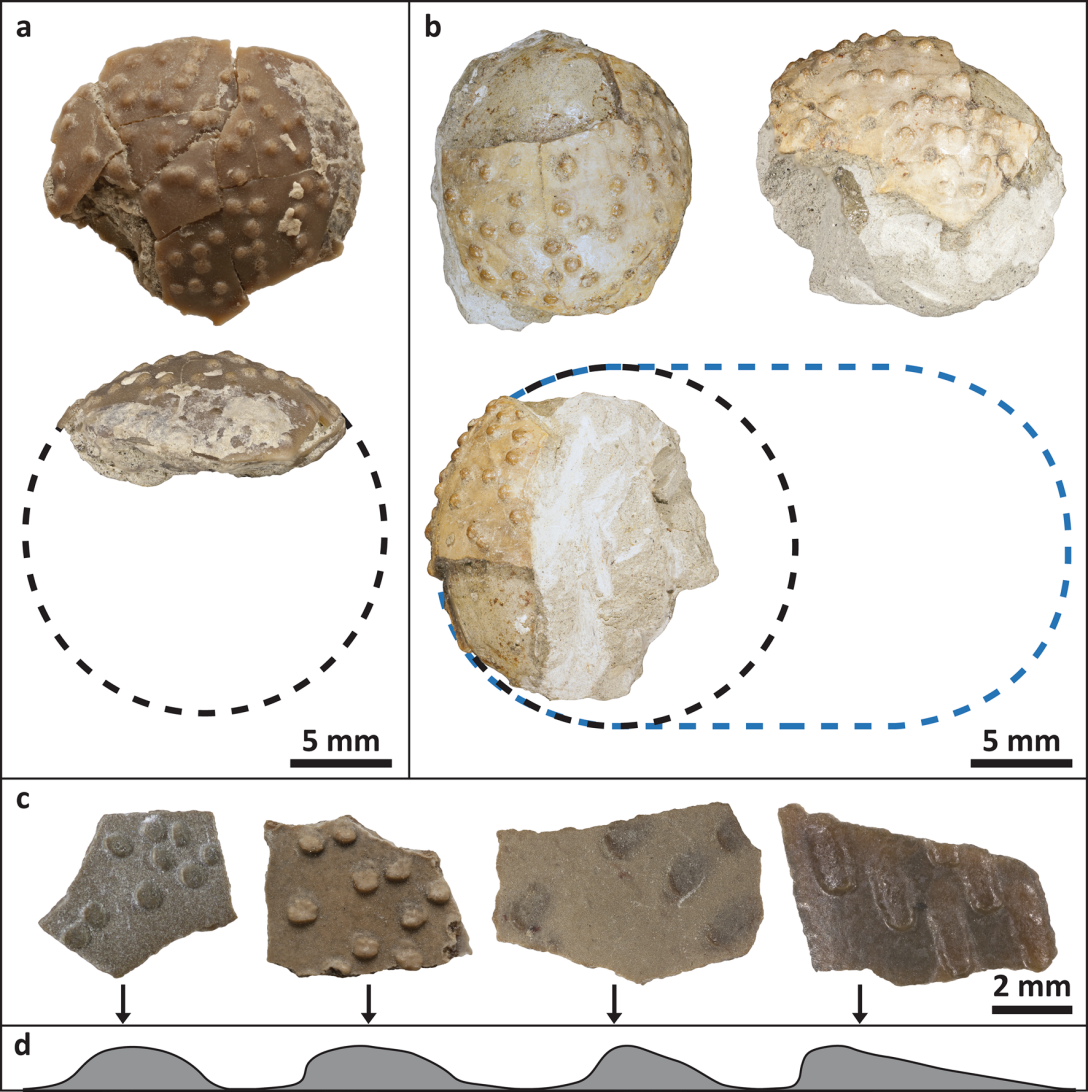
*Stillatuberoolithus storrsi* oogen. et oosp. nov. partial eggs and eggshell. (**a**) Partial egg (DMNH EPV.65736). Dotted circle indicates estimated spherical egg size based upon curvature (**b**) Partial egg (DMNH EPV.128286, paratype). Note circular dispersituberculate nodes visible in apex view (top left) and slightly elongated nodes with long axes aligned visible in lateral view (top right). Dotted circle indicates estimated spherical egg size based upon curvature. Blue dotted line indicates potential ellipsoidal egg size (profile is based upon laevisoolithid eggs); (**c)** Eggshell fragments (left to right, UCM 1039A, DMNH EPV.65602 holotype, UCM 1139 paratype, UCM 1083) displaying variation in ornamentation grading from circular to oval-shaped, flattened nodes that taper in cross section; (**d**) Node profiles grading from circular (left) to oval-shaped (right).

**Oogeneric diagnosis.** As for the type and only oospecies.

**Stratigraphic and geographic distribution.** Upper Cretaceous (Campanian). Kaiparowits Formation of southern Utah.

**Etymology.** From the Latin *stillare*, to drip, in reference to the morphology of the nodes; from the Latin *tuber*, bump, in reference to the nodes; and *oolithus*, combination from the Greek meaning ‘egg stone’ commonly applied to fossil eggshell. The oospecies name honours Dr. Glenn Storrs of the Cincinnati Museum of Natural History for his mentorship in the field.

**Holotype.** DMNH EPV.65602 (Denver Museum of Nature and Science), eggshell fragment and thin sections.

**Paratypes.** DMNH EPV.128286, partial egg; UCM 1139 (University of Colorado Museum of Natural History), eggshell fragments.

**Referred specimens.** DMNH EPV.65670, DMNH EPV.70356, DMNH EPV.87976, DMNH EPV.89797, DMNH EPV.89827, DMNH EPV.89878, UCM 1022, UCM 1047, UCM 1048, UCM 1073, UCM 1082, UCM 1083, UCM 1091, UCM 1092, UCM 1140, UCM 1142 (eggshell) and DMNH EPV.65736 (partial egg).

**Type locality and age.** Denver Museum of Nature and Science (DMNH) localities 4386, 4361 and University of Colorado Museum of Natural History (UCM) locality 2000089 in Garfield and Kane counties, Utah; Upper Cretaceous (Upper Campanian) Kaiparowits Formation.

**Oospecies diagnosis.** Eggs are small (partial eggs indicate 17.7 mm diameter); eggshell displays dispersituberculate ornamentation consisting of irregularly spaced, flattened nodes which grade from circular to oval in plan view (with long axes of oval nodes aligned) over the surface of the egg; eggshell thickness ranging from 0.31 to 0.40 mm (0.31–0.59 mm including ornamentation); two structural layers of calcite include the mammillary layer (ML) and continuous layer (CL) which are delineated by an abrupt, linear boundary; ML:CL thickness ratio of 1:1.1–1:1.5; pores are funnel-shaped through the mammillary and continuous layers, increasing in diameter from the interior of the eggshell towards the surface and branching in the nodes; pore openings at the surface are small (0.03–0.04 mm) and located on the nodes; nodes are crystallographically discontinuous from the CL.

**Description.** *Stillatuberoolithus storrsi* is represented by two partial eggs and 29 eggshell fragments from 12 localities. The partial eggs are small (12–16 mm) and, based upon curvature, had an estimated diameter of 17.7 mm (Fig. 2a,b). The partial eggs appear spherical with some lithostatic compression, though they may also represent the poles of ellipsoidal eggs. More complete specimens would be required to confirm precise egg size and shape. Dispersituberculate ornamentation consists of isolated, flattened nodes which grade from circular to oval in plan view over the surface of the egg, with long axes of oval nodes aligned (Fig. 2c,d). Oval nodes may also overlap one another. The partial egg DMNH EPV.128286 has circular nodes at its apex and oval nodes along the periphery, with long axes aligned towards the apex.

**Figure 3:**
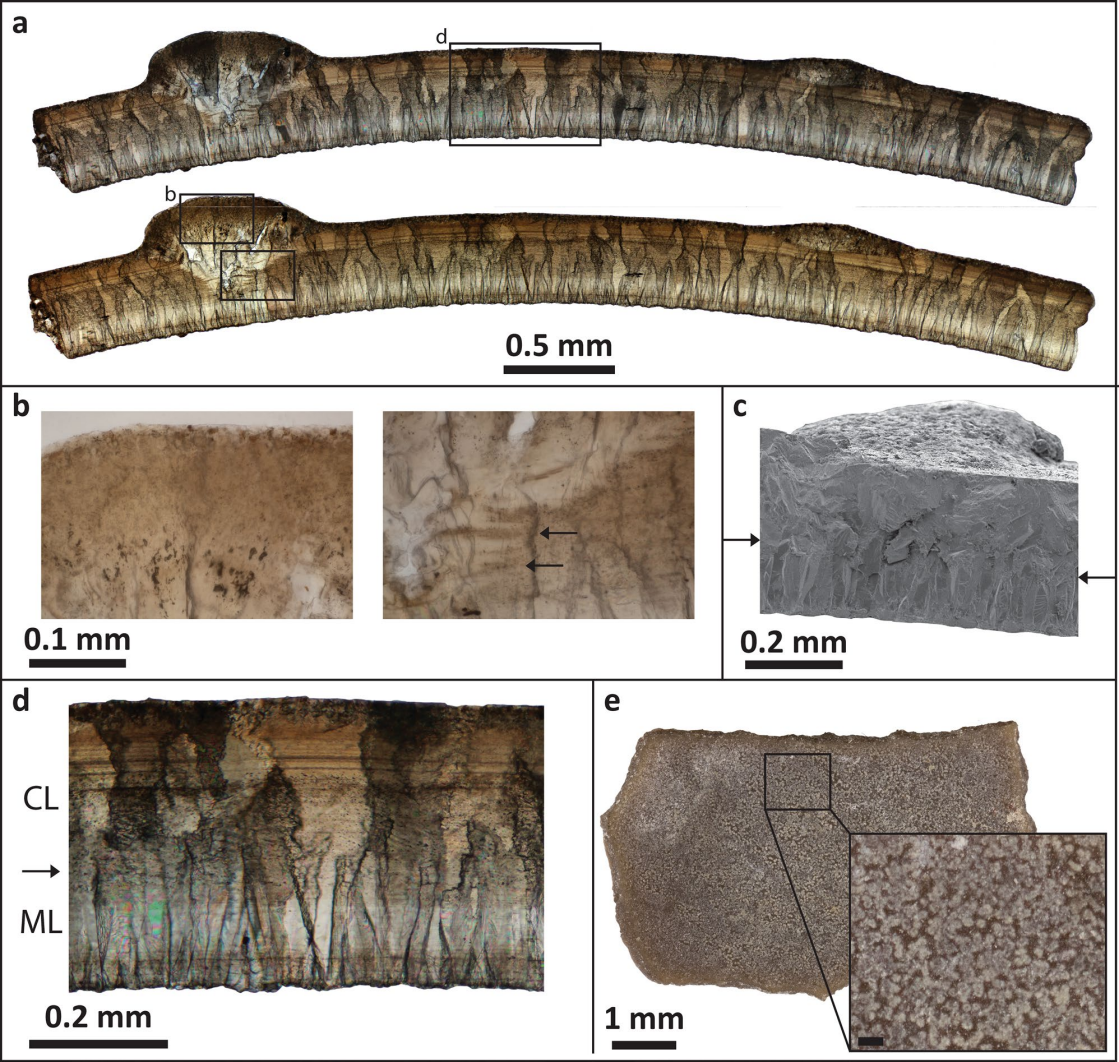
*Stillatuberoolithus storrsi* oogen. et oosp. nov. microscopic imagery (**a**) Radial thin section of DMNH EPV. 65602 (holotype) in cross-polarized (top) and plane light (bottom). Note columnar extinction pattern with irregular boundaries in the continuous layer. Compare to Sahni et al. (1994) Fig. 13.10d and Tanaka et al.^18^ Fig. 2e; (**b**) Insets from the node in section A showing that accretion lines are absent through the nodes (left). Accretion lines (arrows) dip downward at the cavities under the nodes, consistent with pore canal systems (right); (**c**) SEM image of UCM 1139 (paratype) in radial view. Arrows indicate boundary between mammillary and continuous layers; (**d**) Right inset from section A providing a close view of eggshell microstructure. Note tightly packed mammillae with tabular ultrastructure and faint “columnar structures” in the continuous layer. Compare to Sahni et al.^19^ Fig. 13.10e. Arrow indicates transition between mammillary and continuous layers; (**e**) Inner surface of UCM 1139 (paratype) displaying tightly packed mammillae. Inset scale is 0.1 mm.

**Figure 4:**
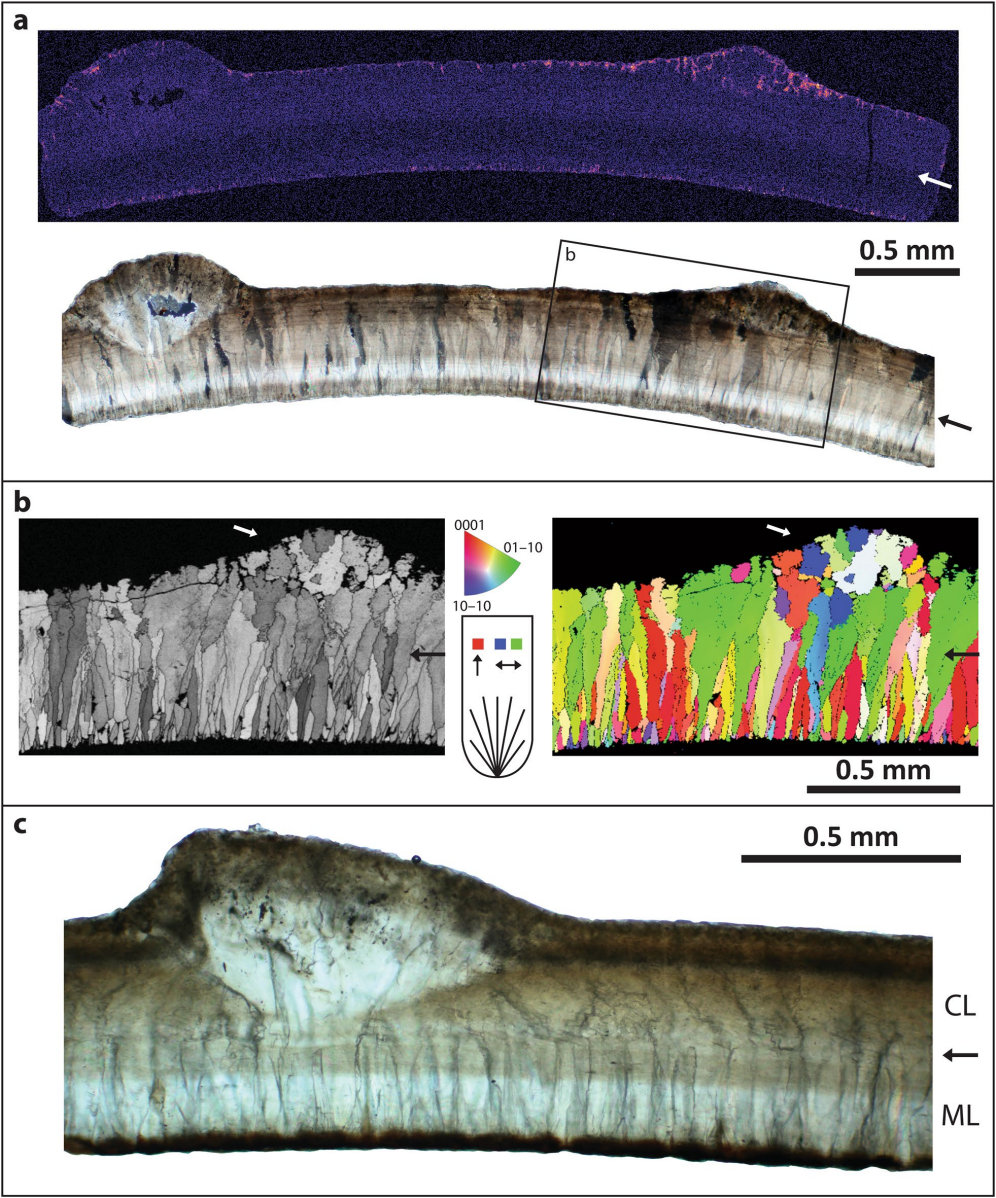
*Stillatuberoolithus storrsi* oogen. et oosp. nov. UCM 1139 (paratype). (**a**) EMPA elemental mapping of magnesium (Mg) through a thin section of eggshell (pictured beneath in cross-polarized light). Colour scale ranges from high (bright magenta) to low (dark magenta) concentration. Lowest concentration is in the mid-upper mammillary layer, with upper boundary distinctly visible (arrow). Pseudocolour was applied to the original grey scale image with Fire LUT in Image J (https://imagej.nih.gov/ij/), and adjustments were applied to the whole image ; (**b**) EBSD band contrast map (left) and IPF Y crystallographic orientation map (right) showing mammillary-continuous layer transition (black arrow) with rugged grain boundaries in the continuous layer. Note the node (white arrow) is crystallographically discontinuous from the underlying eggshell. Key indicates calcite crystal c-axis orientation; (**c**) Radial thin section in plane light displaying the boundary between the mammillary and continuous layers (arrow). Note rugged grain boundaries in the continuous layer.

Eggshell consists of two structural layers of calcite separated by a discrete boundary (Figs. 3, 4). The inner mammillary layer (ML) is characterized by tabular ultrastructure with tightly spaced mammillae (Figs. 3d,e). The continuous layer (CL) includes horizontal accretion lines and displays irregular grain boundaries extending to the eggshell surface with a columnar extinction pattern under cross-polarized light. Eggshell is 0.31–0.40 mm-thick (0.31–0.59 mm including ornamentation) with a ML:CL thickness ratio of 1:1.1–1:1.5 (excluding ornamentation). Accretion lines are not traceable near nodes and do not arch below nodes to follow surface of eggshell (Fig. 3b). Electron backscatter diffraction (EBSD) Inverse Pole Figure (IPF) maps demonstrate that the nodes are crystallographically discontinuous from the underlying eggshell; crystals comprising the node originate within the CL and splay outward in a wedge shape to form the node (Fig. 4b). Electron microprobe analysis (EMPA) elemental mapping indicates that the concentration of magnesium is higher along the inner and outer surfaces, and reaches a minimum between the middle and outer edge of the ML (Fig. 4a). EMPA analyses of calcium, phosphorus, and sulphur did not reveal any patterns.

**Figure 5:**
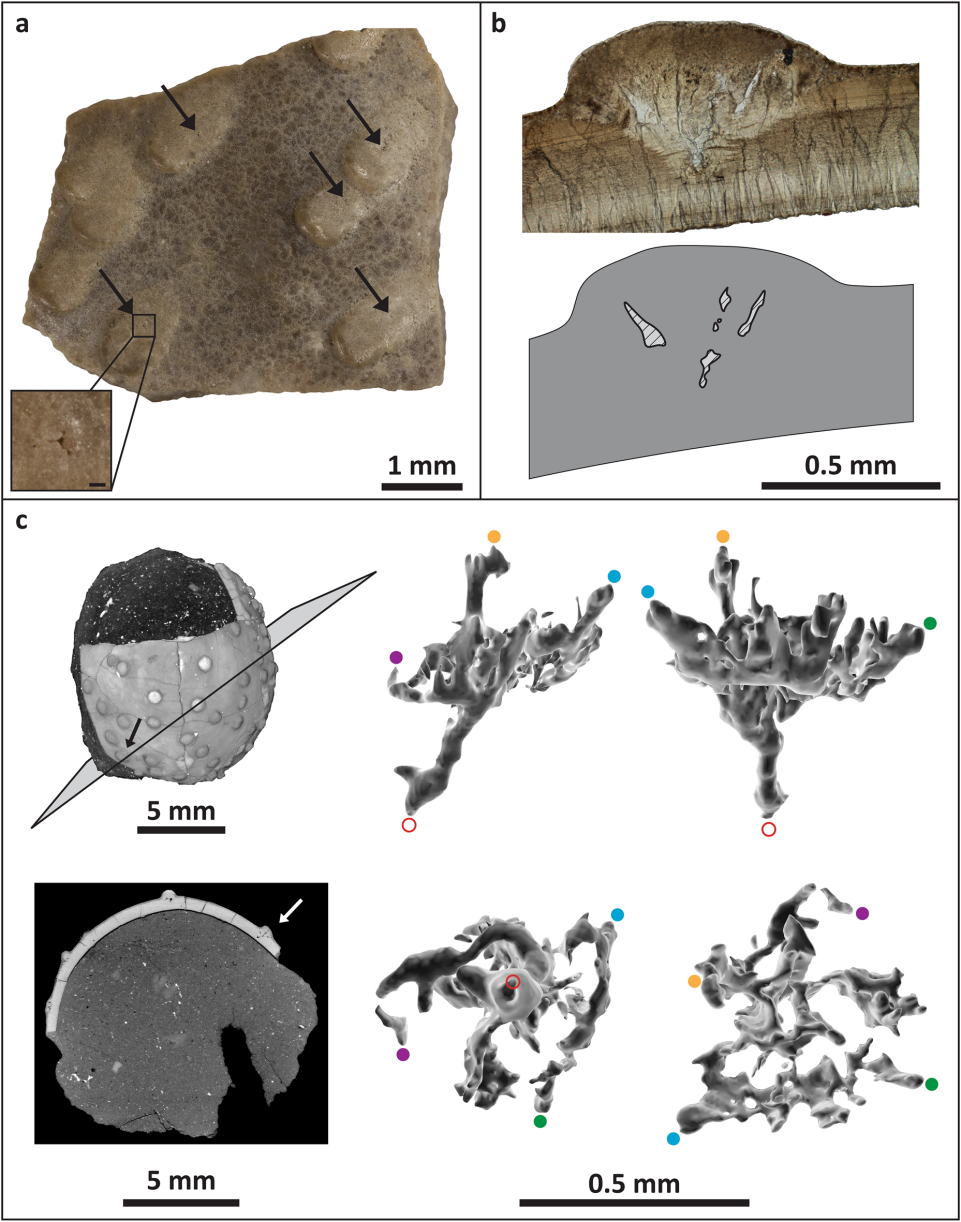
*Stillatuberoolithus storrsi* oogen. et oosp. nov. pore structure. (**a**) Outer surface of UCM 1073 showing dispersituberculate ornamentation with slightly elongated nodes. Note very small pore openings on the nodes (arrows). Inset shows pore opening with a scale of 0.2 mm; (**b**) Radial thin section of DMNH EPV.65602 (holotype) with diagram highlighting open cavities within the node due to branching pores; (**c**) Rendered CT scans of partial egg (DMNH EPV.128286, paratype). Pore canals are evident within nodes; straight, perpendicular lines in shell between nodes are fractures in the eggshell. A pore canal within a node free of cracks was selected for partitioning (arrow). Four 3D views of the selected pore canal are provided. Upper left and upper right images are lateral views. Lower left image is a view from the base of the pore canal, looking up towards the outer eggshell surface. Lower right image is a view from the top of the pore canal, looking down towards the inner surface of the eggshell. Red open circle indicates where the pore canal meets the inner surface of the eggshell. Orange and blue dots indicate where the pore reaches the outer surface of the node. Purple and green dots provided to assist with orientation. Images in (**c**) generated with Dragonfly software, Version 4.1.0.647 for Windows; software from Object Research Systems (ORS) Inc., Montreal, Canada is available at http://theobjects.com/dragonfly.

Pore openings are located on the nodes (Fig. 5a) and are not readily visible on the surface due to their small size (0.03–0.04 mm diameter). Pore structure in *S. storrsi* is difficult to characterize owing to the three-dimensional nature of the pore canals, which make them difficult to observe in two-dimensional views, including traditional thin sections (Fig. 5b). Cross sections of pores in radial thin sections appear as cavities under the nodes (Figs. 3f, 5b). Accretion lines dip at these cavities, indicating that they are biogenic structures that were part of the pore canal system (Fig. 3b). Multiple thin sections through the same pore show that canals are sinuous, increasing in diameter away from the mammillary layer and branching towards the surface within the nodes. X-ray computed tomography (CT) scans of the partial egg specimen DMNH EPV.128286 allows for three-dimensional visualization of pore spaces, revealing that the pore canals branch in a complex anastomosing system within each node (Fig. 5c). Branches show a high degree of interconnectivity. Many of the branches are difficult to trace as they approach the outer surface of the eggshell and appear to pinch out before they reach the surface.

## Discussion

Eggshell has been traditionally organized into structural morphotypes; however, this system is being largely supplanted by the use of parataxonomic binomial nomenclature^5,20^. Preliminary description of *S. storrsi* placed it within the oofamily Laevisoolithidae based upon eggshell thickness, ornamentation, and egg size, as well as its close microstructural similarity to the laevisoolithid taxon *Subtiliolithus kachchhensis*^4^. Additional imagery has allowed better characterization of the unique pore morphology of *S. storrsi*, further distinguishing the oospecies from other laevisoolithid taxa and warranting exclusion from the oofamily as it is presently defined. Further, certain taxa placed within Laevisoolithidae (discussed briefly below) currently strain the diagnosis of the oofamily such that a re-evaluation of the taxonomic group utilizing updated methods is in order, though outside the scope of this treatment. However, because of the similarity of *S. storrsi* to this oofamily, we include brief descriptions and comparisons of laevisoolithid ootaxa.

Ootaxa placed within Laevisoolithidae have previously been described from the Upper Cretaceous of India, Mongolia, Morocco, as well as the Lower Cretaceous of Japan and include *Laevisoolithus sochavi*, *Subtiliolithus microtuberculatus*, *Subtiliolithus kachchhensis*, *Subtiliolithus hyogoensis*, and *Tipoolithus achloujensis* (Table 1)^21–25^. Unassigned laevisoolithid eggshell has also been described from the Upper Cretaceous of France^26^. In the traditional classification system, the oofamily Laevisoolithidae was considered part of the Ornithoid Basic (Ratite) morphotype which included eggshells with two microstructural layers (mammillary and continuous)^21,22^. It is characterized by small, ellipsoid eggs with smooth, thin eggshell (0.3–0.6 mm), a mammillary layer one half to two thirds the eggshell thickness (ML:CL thickness ratio of 1:1–2:1), and angusticanaliculate pores. At the time of its characterization, Mikhailov^21^ included branching morphology in the definition of ‘angusticanaliculate’. In subsequent publications, however, ‘angusticanaliculate’ refers to straight, unbranching pores^22^. In addition, when Subtiliolithidae became a junior synonym to Laevisoolithidae, it included taxa which did not fit within the revised diagnosis^22^. For example, the diagnosis specifies smooth eggshell but includes the ornamented *Subtiliolithus microtuberculatus* and *Subtiliolithus kachchhensis*. In addition, the ML:CL ratios of the included taxa exceed the diagnosed range of 1:1–2:1 (Table 1).

**Table 1:** Comparison of *Stillatuberoolithus storrsi,* oogen. et oosp. nov. to similar ootaxa. Brackets indicate eggshell thicknesses including ornamentation.

Ootaxon	Age	Formation	Location	Eggshell thickness (mm)	ML:CL	Ornamentation	Reference
*Stillatuberoolithus storrsi,* oogen. et oosp. nov	Campanian	Kaiparowits	Utah	0.31–0.4 [0.37–0.59]	1:1.1–1:1.5	Dispersituberculate with flat nodes	This paper; Holotype
Laevisoolithidae (diagnosis)				0.3–0.6	2:1–1:1	Smooth	^22^
*Subtiliolithus kachchhensis*	Maastrichtian	Lameta	India	[0.35–0.5]	1:1–1:2	Dispersituberculate	^19,23^
*Subtiliolithus kachchhensis* (revised)	Maastrichtian	Lameta	India	0.467–0.491	1:1–1:2	Dispersituberculate	^27^
*Subtiliolithus microtuberculatus*	Maastrichtian	Nemegt	Mongolia	0.3–0.4	3:1–2:1	Very fine tubercles	^21^
*Subtiliollithus hyogoensis*	Albian	Ohyamashimo	Japan	0.22–0.41	1:0.9–1:1.3	Dispersituberculate	^25^
*Laevisoolithus sochavai*	Campanian–Maastrichtian	Djadokhta & Nemegt	Mongolia	< 1.0	2:1–1:1	Smooth	^21^
*Tipoolithus achloujensis*	Maastrichtian	Irbzer	Morocco	0.4–0.65	1:1–1:2	Dispersituberculate	^24^
Laevisoolithidae	Maastrichtian		France	0.25–0.37	1:2.5–1:3	Smooth	^26^
*Porituberoolithus warnerensis*	Campanian	Kaiparowits	Utah	0.51–0.68 [0.57–0.91]	1:2	Dispersituberculate	^4^
*P. warnerensis*	Campanian	Oldman	Alberta	0.5–0.65	1:2	Dispersituberculate	^28^; Holotype
*P. warnerensis*	Campanian	Dinosaur Park & Oldman	Alberta			Disperistuberculate & Flat nodes	^29^
*P. warnerensis*	Maastrichtian	Willow Creek	Alberta	0.45–0.78 [0.72–1.12]	1:1–1:2	Disperistuberculate & Flat nodes	^30^
*P. warnerensis* cf	Santonian	Milk river	Alberta	0.37 [0.71]			^31^
*Porituberoolithus* sp.	Campanian	Fruitland	New Mexico	0.31–0.72	1:2	Disperistuberculate & Flat nodes	^18^
cf. *Porituberoolithus*	Campanian	Aguja	Texas	[0.57–0.73]	1:2	Flat nodes	^32^
*Pseudogeckoolithus nodosus*	Maastrichtian	Tremp	Spain	0.3–0.35	1:7–1:9	Dispersituberculate	^33^
*Pseudogeckoolithus* cf. *nodosus*	Maastrichtian	Densus-Ciula Arén	Romania Spain	0.28 [0.35]	1:3–1:9	Dispersituberculate	^34^
*Pseudogeckoolithus* aff. *tirboulensis*	Santonian Campanian Maastrichtian	Csehbánya Sebes Pui beds	Hungary Romania	0.13–0.22 [0.2–0.25]	1:1–1:6	Dispersituberculate	^34^
*Dimorphoolithus bennetti*	Maastrichtian	Hell Creek	Montana	[0.561–0.850]	1:2.5	Bimodal dispersituberculate	^35^
Anguimorph	Early Cretaceous		Thailand	[0.446–0.537]	n/a	Dispersituberculate	^36,37^

*Stillatuberoolithus storrsi* is most similar to eggshell from the Maastrichtian Lameta Formation of India which was initially referred to as “Ornithoid type”^19^. Khosla and Sahni^23^ subsequently assigned this eggshell to *Subtiliolithus kachchhensis* within the oofamily Subtiliolithidae (= Laevisoolithidae^22^). *Subtiliolithus kachchhensis* eggshell is two-layered with a distinct boundary between mammillary and continuous layers, 0.35–0.50 mm thick (including ornamentation), displays low, subcircular, irregularly spaced dispersituberculate nodes, shows columnar extinction in the CL, displays tightly spaced mammillae, and has a ML:CL thickness ratio of 1:1–1:2. Like *S. storrsi*, pore openings are so small that they are not readily visible on the eggshell surface and cross sections of the nodes reveal cavities similar to pores of *S. storrsi*, though full pore morphology is hard to discern (see Fig. 13.10c in Sahni et al.^19^). *Stillatuberoolithus storrsi* can be distinguished from *Subtiliolithus kachchhensis* and the oogenus *Subtiliolithus* as a whole on the basis of node morphology (circular and oval nodes vs. subcircular only) and complex, branching pore structure.

The unassigned laevisoolithid eggshell from France and *L. sochavai* differ from *S. storrsi* in that they do not possess ornamentation and have different ML:CL ratios (1:2.5–1:3 and 2:1–1:1, respectively). *Tipoolithus achloujensis* has angusticanaliculate pores with wide pore openings both atop the nodes (similar to *Porituberoolithus warnerensis*) and on the eggshell surface between nodes. *Subtiliolithus microtuberculatus* has a much larger ML:CL thickness ratio (3:1–2:1) than *S. storrsi*. *Subtiliolithus hyogoensis* has conical, tightly spaced, and often coalescing nodes in contrast to the discrete, flattened nodes of *S. storrsi*.

Ootaxa outside Laevisoolithidae bear similarities to *S. storrsi* as well. *Pseudogeckoolithus nodosus* from the Maastrichtian Tremp Formation of Spain also has dispersituberculate ornamentation with pore openings situated atop the nodes and has an overlapping eggshell thickness (0.30–0.35 mm); however, it is unlike *Stillatuberoolithus storrsi* in having a prismatic ultrastructure and a thinner mammillary layer relative to the rest of the eggshell (ML:PL thickness ratio of 1:7–1:9)^33^. *Pseudogeckoolithus* aff. *tirboulensis* from the Late Cretaceous of Hungary and Romania differs from *S. storrsi* in having thinner eggshell and a thinner mammillary layer ratio. In addition, EBSD IPF mapping demonstrates that ornamentation of *Pseudogeckoolithus* eggshell is crystallographically continuous with the underlying eggshell^34^.

Eggs of similar size and shape to *Stillatuberoolithus storrsi* have been described from the Lower Cretaceous of Thailand^36,37^. These 18 × 11 mm eggs were initially attributed to theropods but were subsequently identified as anguimorph lizard based upon embryonic remains within the eggs. The thickness of the eggshell averages 0.35 mm (0.45–0.54 mm including dispersituberculate ornamentation) and has funnel-shaped pore canals opening at the nodes. However, the anguimorph eggshell differs from *S. storrsi* in overall microstructure (a single structural layer), pore geometry (unbranched with the wide end of the funnel at the inner surface of the eggshell, resulting in depressions on the inner surface), and node morphology (nodes are conical).

Gekkoolithid eggs are typically small (8–20 mm diameter), spheroidal to ellipsoidal, and can display dispersituberculate ornamentation associated with complex pore structures, similar to *S. storrsi*^38^. However, rigid gekkotan eggshell is composed of a single layer of compact columnar calcite crystals ranging from 0.04 to 0.20 mm thick, whereas *S. storrsi* eggshell is thicker and displays both ML and CL layers. Pore canals in modern *Gekko gecko* eggshells are retecanaliculate, consisting of a series of labyrinthine channels associated with wedge-shaped concretions that may detach from the inner eggshell surface leaving pits^39^. Moreover, descriptions of branching gekkoolithid pore canals are inversely oriented compared to *S. storrsi*. Non-branching funnel-shaped gekkoolithid pore canals are also oriented in the opposite direction (wider at the inner surface). Where nodes are present in gekkoolithid eggshell, they are composed of randomly oriented calcite grains continuous with the underlying eggshell (contrary to *S. storrsi*)^34,39,40^. No descriptions of gekkoolithid eggshell indicate funnel-shaped pore canals which are thinnest at the inner surface, branching into a crystallographically distinct wedge composed of radiating calcite (characteristic of *S. storrsi*)*.*

*Dimorphoolithus bennetti* (oofamily Tubercuoolithidae) from the Maastrichtian Hell Creek Formation of Montana also includes pore openings atop nodes. It differs from *Stillatuberoolithus storrsi* in that it is thicker (0.56–0.85 mm, including ornamentation), displays bimodal dispersituberculate ornamentation consisting of broad tubercles as well as smaller conical nodes, has funnel-shaped pore openings that can occur between nodes, and has a smaller ML:CL thickness ratio (1:2.5)^35^.

**Figure 6:**
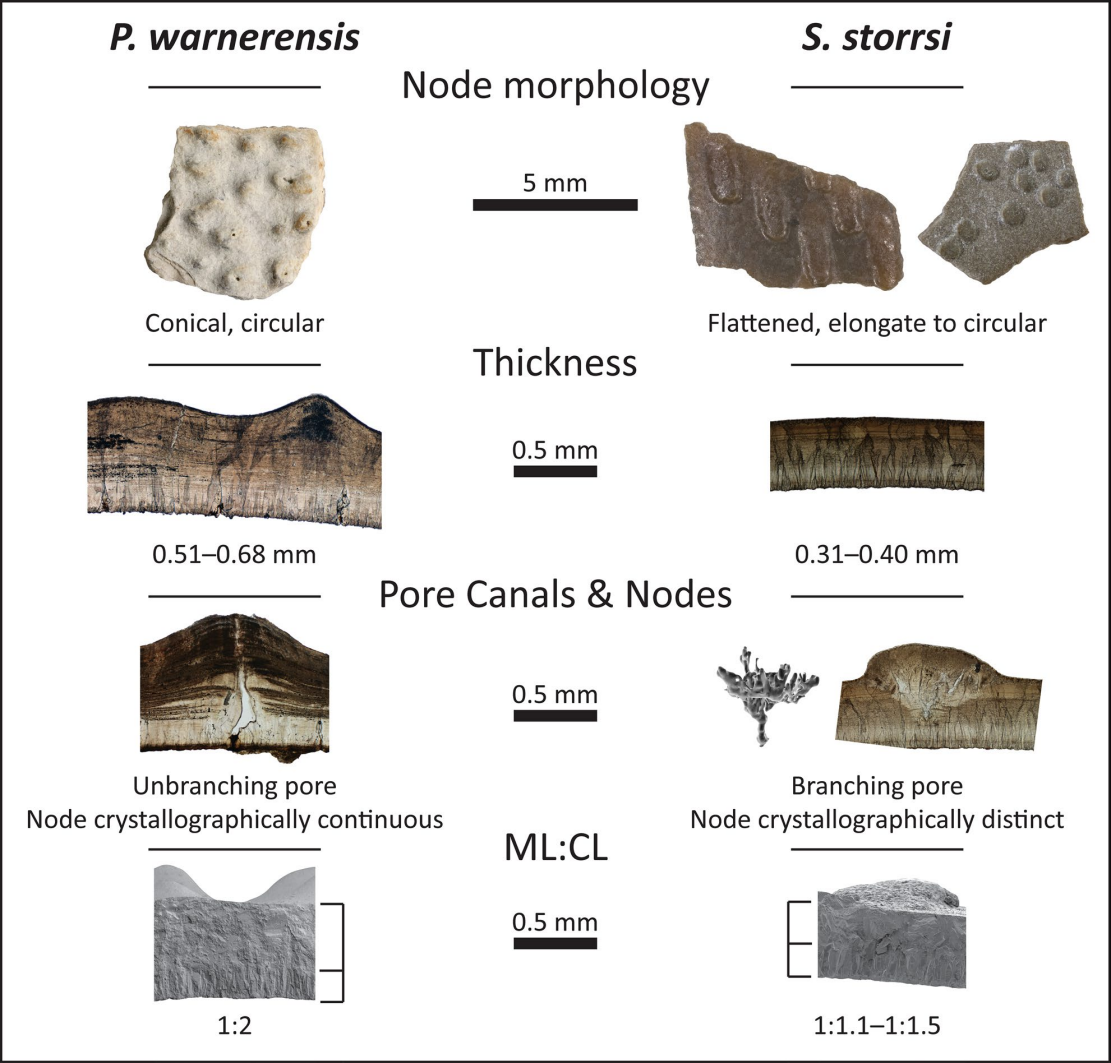
Comparison of *Porituberoolithus warnerensis* and *Stillatuberoolithus storrsi* oogen. et oosp. nov. from the Kaiparowits Formation. *P. warnerensis* specimen numbers from the top-down: DMNH EPV.64663, UCM 1051, DMNH EPV.64663, UCM 1051. *S. storrsi* specimen numbers cited in previous figures.

Previous studies of Campanian eggshell from the Western Interior have referred eggshell similar to *S. storrsi* to *Porituberoolithus warnerensis*^18,29,32^. Zelenitsky et al.^28^ described the oospecies *Porituberoolithus warnerensis* (incertae sedis) from the Campanian Oldman Formation of southern Alberta, Canada; this oospecies is diagnosed as possessing an ornithoid ratite morphotype, with an unbranching angusticanaliculate pore system, pore openings situated on nodes, dispersituberculate ornamentation, eggshell thickness 0.50–0.65 mm, and an ML:CL thickness ratio of 1:2. Zelenitsky and Sloboda^29^ later amended the original diagnosis of *P. warnerensis* to also include “distinctly flattened nodes,” but the paper does not include images of thin sections that allow comparisons of microstructure between the eggshells with flattened nodes to the initial description of *P. warnerensis* with conical nodes. Subsequent publications have grouped eggshell with pore openings atop flattened nodes into *Porituberoolithus* despite the fact that the eggshell differs microstructurally from the oogenus. For example, Tanaka et al.^18^ describe a suite of eggshell identified as *Porituberoolithus* sp. from the Campanian Fruitland Formation of north-western New Mexico that appears to include both *P. warnerensis* (NMMNH P-38474a, see Tanaka et al.^18^ Fig. 2c) and *S. storrsi* (NMMNH P-38025a, see Tanaka et al.^18^ Fig. 2e). In addition, Welsh and Sankey^32^ describe cf. *Porituberoolithus* eggshell from the Campanian Aguja Formation of Texas that is comparable to *S. storrsi*. Eggshell with flattened nodes from the Kaiparowits Formation, here assigned to *Stillatuberoolithus storrsi* oogen. et oosp. nov., differs from the original diagnosis of *P. warnerensis* in overall thickness (0.31–0.41 mm compared to 0.50–0.65 mm), the ML:CL thickness ratio (1:1.1–1:1.5 vs. 1:2), pore structure and size, and the morphology and microstructure of the nodes (Fig. 6; Table 1). No partial or complete eggs have been described for *P. warnerensis* that can be compared. In contrast to *S. storrsi*, the pores of *P. warnerensis* are unbranching and have larger (0.06–0.13 mm in diameter) pore openings. The nodes of most ornamented eggshell (e.g. Elongatoolithidae, Spheroolithidae, *P. warnerensis*) are extensions of the eggshell beneath, demonstrating crystallographic continuity with accretion lines paralleling the surface of the eggshell^34,41^. In contrast, the nodes of *S. storrsi* interrupt accretion lines and are crystallographically distinct from the underlying eggshell (Fig. 4), originating within the CL and splaying outward in a wedge shape to form the node (similar to *Dromaius*). Choi et al.^34^ suggest that distinct mechanisms of ornamentation construction account for these differences in crystal structure. The nodes of *S. storrsi* are distinct both morphologically and microstructurally from *P. warnerensis* (Fig. 6)*.* It is unlikely that these differences are attributable to diagenesis alone, since the features outlined above recur across multiple specimens, localities, and (likely) geologic formations.

It is more parsimonious to assign the Kaiparowits eggshell to a new oogenus and oospecies than it would be to amend the oogenus *Porituberoolithus* in almost every aspect to accommodate the Kaiparowits eggshell. Based upon published images, eggshell comparable to *S. storrsi* likely also occur in the Campanian Fruitland, Aguja, and Dinosaur Park formations of New Mexico, Texas, and Alberta, respectively^18,29,32^. However, we refrain from formally reassigning other described eggshell to *S. storrsi* without examining thin sections.

Pore canal morphology is a key diagnostic feature in fossil eggshell classification, and is primarily characterized utilizing thin sections^21^. While two-dimensional analysis is adequate for determining the shape of most pore canals, it offers limited information when applied to more complex pore systems. Thin sections of *S. storrsi* hint at the intricate pore structure within each node, and display disconnected cavities that suggest a branching morphology. However, the thin sections do not show the complex, anastomosing morphology evident in CT scans of *S. storrsi*. X-ray computed tomography scanning has been increasingly utilized as a high-resolution, non-destructive method for eggshell characterization with a number of applications^42,43^.

**Figure 7:**
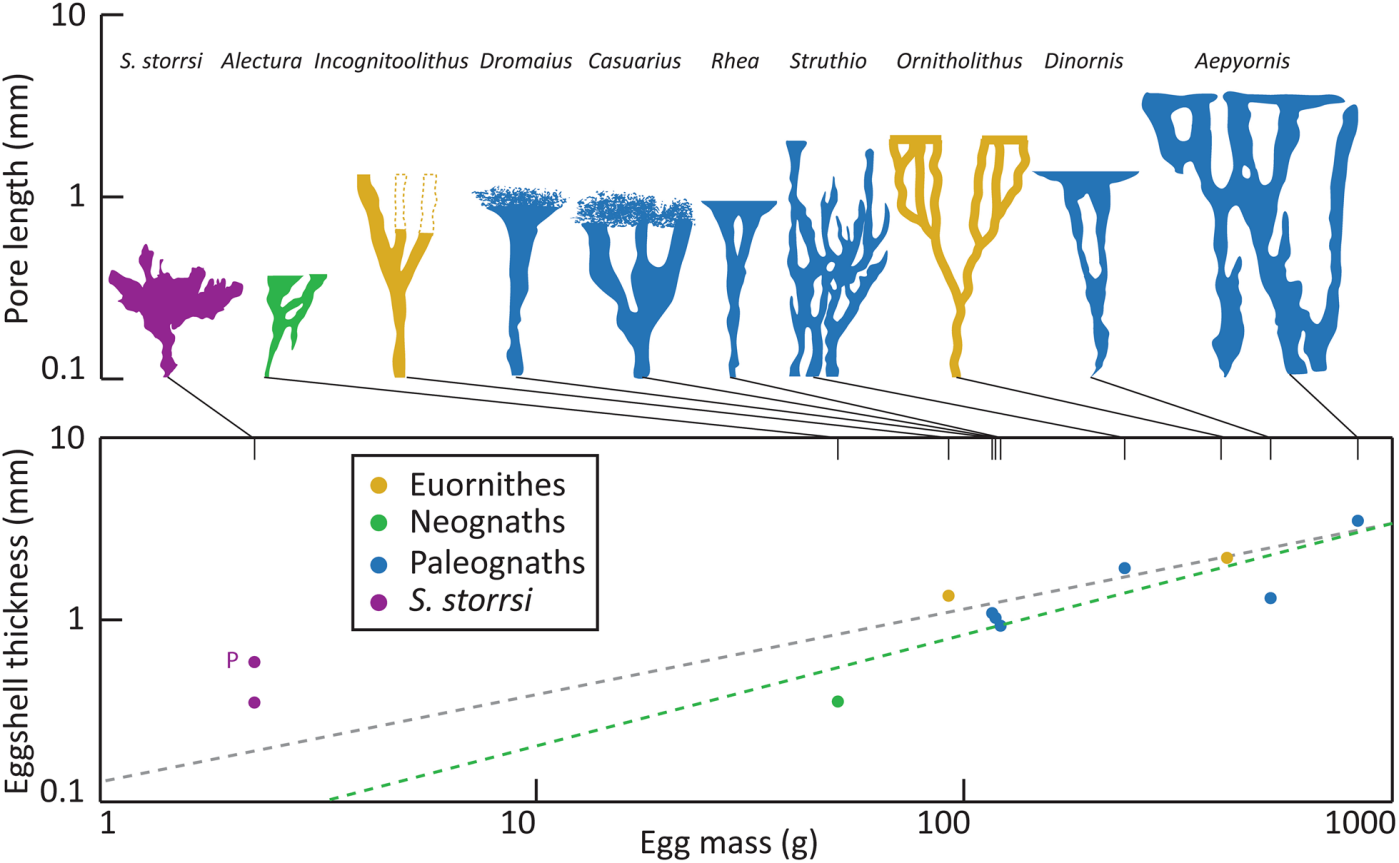
Comparison of branching pore canal morphology with pore length (equivalent to eggshell thickness) and egg mass plotted on logarithmic scales. In the case of *S. storrsi*, pore length is not equivalent to average eggshell thickness since the pores occur within nodes (point P is the eggshell thickness at the pores/nodes). Green dashed line is avian regression from Ar et al.^51^ (N = 367). Grey dashed line is non-avian theropod regression from Deeming^52^ (N = 11). Pore silhouettes, pore length values, and egg mass values from various publications^5,44–48,51,53,54^. Egg mass estimates for fossil taxa calculated using equation from Hoyt^55^.

Branching pores of varying morphologies occur in the eggshell of several extant and extinct avian taxa, especially within palaeognath birds (Fig. 7). The pore canals of *Rhea*, *Dinornis*, and *Aepyornis* eggshells display relatively simple morphologies that branch within one plane, appearing at the surface as linear arrangements of pore openings, similar to *Ornitholithus* and *Incognitoolithus*^44−47^. The branches of pores canals within megapode eggs (*Leipoa ocellata* and *Alectura lathami*) are laterally connected by channels running parallel to the eggshell surface^48^. *Casuarius* and *Dromaius* pore canals are either unbranched or split into a limited number of branches that terminate in complex reticulate structures at the eggshell surface^44,49^. In contrast, *Struthio* displays complex, interconnected pore canal systems with some branches terminating before reaching the surface, similar to *S. storrsi*^44,50^.

Several functional explanations have been suggested for the different branching pore morphologies outlined here. Considering that eggshell thickness is inversely proportional to gas conductance^51^, Tullet and Board^56^ and Tullet^57^ suggested that the branching pores of *Struthio* eggs allow for increased diffusion of respiratory gases, noting that the large eggs of palaeognath birds may require branching pore canals to compensate for longer pore length through thicker eggshell and to overcome the larger surface area to volume ratio. It seems unlikely that this functionality would apply to *S. storrsi* given the small size of the egg; however, when compared to regression lines of eggshell thickness versus egg mass in avian and non-avian theropods, the eggshell thickness of *S. storrsi* is considerable for such a small egg and is even thicker through the nodes where the pores are located (Julia Clarke & Lucas Legendre pers. comm.). Thus, in *S. storrsi,* the branching pores may facilitate diffusion across its (relatively) thick eggshell (Fig. 7). Grellet-Tinner et al.^48^ suggested that the interconnected canals of megapode birds would allow for lateral diffusion of gases if a pore opening became obstructed, a useful adaptation for eggs that are incubated within buried nests. Determination of the eggshell gas conductance of *S. storrsi* would allow for interpretations of nesting environment, but accurate assessment requires more complete specimens and awaits additional finds.

The small size and two-layered microstructure of *S. storrsi* eggshell is consistent with eggs laid by small-bodied avian or non-avian theropods. The continuous layer displays columnar extinction with irregular crystal boundaries typical of theropod eggshell^58^. The mammillary layer can be distinguished in thin section, SEM imagery, and EBSD crystallographic orientation mapping. Notably, a high proportion of calcite grains in *S. storrsi* have a horizontal c-axis (indicated by blue or green colours in the IPF Y map; Fig. 4b), which is typical of neognath eggshells^34,41,58^. In contrast, other analysed Cretaceous theropod eggshells show calcite grains with a vertically-laid c-axis, and appear reddish in IPF Y maps^34,41,58^. EMPA elemental mapping reveals higher concentrations of magnesium along the inner and outer eggshell surfaces with a minimum concentration near the ML/CL transition, a pattern observed in avian eggshell^59^. When compared to regression lines of eggshell thickness versus egg mass, the values for *S. storrsi* are more similar to non-avian theropod than to avian eggs (Fig. 7); however, deducing phylogenetic signals in these egg characters is a matter of current study (Julia Clarke & Lucas Legendre pers. comm.). Small-bodied theropods and enantiornithine birds are preserved within the Kaiparowits Formation and represent potential egg-laying taxa; however, assignment of *S. storrsi* to a skeletal taxon awaits discovery of embryonic remains.

The Kaiparowits Formation partial eggs have an estimated diameter of approximately 17.7 mm, assuming a spherical shape. The eggs may have been spherical or oblong in morphology, but regardless of shape, they are among the smallest Mesozoic eggs described. The closest comparably small fossil vertebrate eggs include a Campanian anguimorph lizard at 18 × 11 mm^36,37^, *Gobioolithus minor* eggs attributed to an enantiornithine at 20–24 × 30–46 mm^60,61^, an avian egg from Mongolia at 15.88 × 25.8 mm^62^, and an avian egg from Brazil at 19.5 × 31.4 mm^63^. The relative rarity of small eggs from other formations may reflect the low preservation potential of small eggs with comparatively thin eggshell. Preservation of eggshell is affected by many factors, including nest site selection (nesting in areas conducive to fossilization), nesting behaviour (open vs. enclosed nests), neonate development (precocial vs. altricial), eggshell structure (soft vs. hard shelled; thin vs. thick eggshell), depositional environment (fine vs. coarse grained sediments), and paleoenvironment (humid/reducing vs. dry/oxidizing)^64–69^. The high sedimentation rate (estimated 41 cm/ka^7^) of the Kaiparowits Formation may have facilitated preservation of the small *S. storrsi* eggs.

*Stillatuberoolithus storrsi* is a remarkably small oospecies that displays a unique suite of features common in both avian and non-avian egg taxa. Jackson et al.^5^ note that no single egg feature unambiguously separates avian from non-avian theropods. Rather, eggs preserve a suite of characters that mirrors the mosaic of behavioural and morphological adaptations characterizing the period of transition which saw the rise of the avian theropod crown group (Table 2). Previous studies have highlighted typically-avian characters and reproductive behaviours observed in some maniraptoran theropods (e.g., asymmetrical eggs, smooth eggshell, 3-layer microstructure, brooding, monoautochronic ovulation) as well as typically-maniraptoran theropod features observed in some early avian eggs (e.g., ornamentation, two-layer microstructure) and note that some features arise and are lost multiple times^5,20,70–73^. The specific combination of small egg size, branching pores, two-layered microstructure, and dispersituberculate ornamentation in the partial eggs described in this study is unique. The tiny, exquisitely preserved, ornamented partial eggs from the Kaiparowits Formation of Grand Staircase-Escalante National Monument not only increase the rich diversity of North American fossil ootaxa but add to our understanding of evolutionary changes reflected in theropod eggs.

**Table 2:** Mosaic of egg features that characterize Cretaceous-Cenozoic avian and non-avian theropod eggs.

Ootaxon (Taxon)	Pores	Egg shape	Egg size (mm)	Eggshell thickness (mm)	Ornamentation	# layers	Reference
*Elongatoolithus* (oviraptorosaur)	Non-branching	Elongate, asymmetrical	70–172 × 15–82	[0.3–1.5]	Yes	2	^74,75^
*Prismatoolithus levis* (*Troodon*)	Non-branching	Elongate, asymmetrical	120–160 × 30–70	0.7–1.28	No	2–3	^76,77^
*Triprismatoolithus*	Non-branching	Elongate, symmetrical	75 × 30	0.53–0.85	Yes	3	^78^
*Dispersituberoolithus*	Non-branching			0.26–0.28	Yes	3	^28^
*Tristraguloolithus*	Non-branching			0.32–0.36	Yes	3	^28^
*Stillatuberoolithus storrsi*	Branching		~ 17.7	0.31–0.4 [0.37–0.59]	Yes	2	This paper
*Himeoolithus murakamii*		Elongate, Asymmetrical	45 × 20	0.15	No	2	^25^
*Styloolithus sabathi* (enantiornithine)		Elongate, asymmetrical	70 × 32	0.25	No	3	^79,80^
*Gobioolithus minor* (enantiornithine)	Non-branching	Elongate, asymmetrical	30–46 × 20–24	0.1–0.2	No	2?	^60,61^
Unassigned (enantiornithine)		Elongate, symmetrical	47.5 × 22.3	0.18	Yes	3	^81,82^
Unassigned (enantiornithine)		Asymmetrical	45 × 27	0.26	No	3	^83^
*Parvoolithus tortuosus* (Avian?)	Non-branching	Elongate, asymmetrical	40 × 25	< 0.1	No	3	^22,67^
Unassigned (Avian?)		Elongate	36 × 25	0.44–0.46	No	2	^84^
Unassigned (avian)	Non-branching	Asymmetrical?	31.4 × 19.5	0.126	No	3	^63^
Unassigned (avian)		Elongate	25.8 × 15.88	0.166		3	^62^
*Ornitholithus* (avian)	Branching	Elongate	118 × 150–200 × 400	1–3	Yes	2	^46^
*Incognitoolithus ramotubulus* (avian)	Branching	Elongate	75–90 × 100–120	1.27–1.43	No	2	^45^
*Medioolithus geiseltalensis* (avian)	Non-branching	Spheroidal	90 × 90	0.76–0.97	No	3	^85^
*Metoolithus nebraskensis* (avian)		Spheroidal	45 × 60	0.75–0.9	Yes	3	^5^
*Microolithus wilsoni* (avian)	Non-branching	Spheroidal	30 × 37	0.6	No	3	^5^
(Extant ratites)	Branching	Spheroidal & ellipsoidal	180 × 150, 133 × 90	1.6–2.7, 1.0	Yes & No	3–4	^45^
(Extant Neognathae)	Non-branching	Asymmetrical	varies 50 × 40	varies 0.4	No	3	^5^
(Extant Megapodiidae)	Branching	Asymmetrical, elongate	60 × 91	[0.35–0.36]	Yes	3	^48^

For extant ratites, egg size and eggshell thickness are given for *Struthio* and *Dromaius*. Extant megapode ranges are from *Alectura lathami*. For extant Neognathae, the egg size and shell thickness of *Gallus gallus domesticus* is provided for reference.

## Methods

The twenty *S. storrsi* eggshell fragments housed within the Karl Hirsch Eggshell Collection at the University of Colorado Museum of Natural History were collected by Jeff Eaton of the Utah Museum of Natural History in 1986 from six localities 267–322 m from the base of the Kaiparowits Formation. The nine Denver Museum of Nature and Science specimens of this ootaxon (including both partial eggs) were collected more recently (2011–2014) from six localities. Of the collected specimens, eleven were selected for analyses including imaging, thin sectioning, scanning electron microscopy (SEM), electron microprobe analysis (EMPA), electron backscatter diffraction (EBSD) imaging, and computed tomography (CT) scanning. Prior to analysis, all specimens were serially imaged using a Canon 5D Mark II digital camera with a 65 mm MP-E macro lens mounted on a Visionary Digital P-51 CamLift. Resulting images were combined using Helicon Focus stacking software to generate composite images for each specimen with greater depths of field. Radial thin sections were prepared in-house by embedding specimens in Struers Epofix epoxy and sectioning cured pucks with a Buehler Isomet low-speed saw with a diamond wafering blade. Thin slices were then polished with a Buehler Ecomet II polisher with 600 grit silicon carbide grinding paper and affixed to frosted petrographic slides with Devcon Two Ton epoxy. Each slide was then ground to optical thickness (~ 40 µm) using a Struers RotoPol-35 and Bortys grinding system. Thin sections were examined with a Leica DMR petrographic microscope (with cover slips and Resolve microscope immersion oil) and imaged with an attached Canon 5D Mark II digital camera. A thin section of UCM 1139 was polished with 5 μm silicon dioxide powder followed by a Buehler 0.05 μm alumina suspension for EMPA and EBSD imaging. The specimen was carbon coated before elemental distributions of magnesium, phosphorus, calcium, and sulphur were mapped with a JEOL JXA-8230 electron probe microanalyzer. The maps were made at 15 keV accelerating voltage, a beam current of 10 nA, 2 × 2 μm pixel resolution, and a dwell time of 20 ms per pixel. The carbon coating was polished off the thin section before it was imaged with electron backscatter diffraction (EBSD) on a Hitachi SU3500 SEM at the Colorado Shared Instrumentation in Nanofabrication and Characterization (COSINC) facility in the College of Engineering and Applied Science at the University of Colorado. The EBSD analyses were run with an accelerating voltage of 15.00 kV, a specimen tilt of 70.00°, a hit rate of 70.02%, 12.95 Hz of acquisition, and 2.5 × 2.5 μm pixel resolution. The specimen selected for SEM imaging was manually split in order to provide a freshly fractured surface before being coated with 4 nm of platinum using a sputter coater and imaged with a Hitachi SU3500 SEM at the COSINC facility. ImageJ software was utilized to measure eggshell characters. Specimen DMNH EPV.128286 was also analysed with a North Star Imaging CT scanner at the University of Texas High-Resolution X-ray Computed Tomography Facility. The specimen was imaged at 110 kV for 100 min, generating 1349 slices. Images were rendered in 3D and pore spaces were manually segmented using Dragonfly three-dimensional visualizer software (Object Research Systems, Inc.). The resulting mesh was then exported into MeshLab open-source software for image capture.
